# Evaluation of the Radiolabeled Boronic Acid-Based FAP Inhibitor MIP-1232 for Atherosclerotic Plaque Imaging

**DOI:** 10.3390/molecules20022081

**Published:** 2015-01-27

**Authors:** Romana Meletta, Adrienne Müller Herde, Aristeidis Chiotellis, Malsor Isa, Zoran Rancic, Nicole Borel, Simon M. Ametamey, Stefanie D. Krämer, Roger Schibli

**Affiliations:** 1 Department of Chemistry and Applied Bioscience of ETH Zurich, Center for Radiopharmaceutical Sciences ETH-PSI-USZ, Vladimir-Prelog-Weg 4, 8093 Zurich, Switzerland; E-Mails: romana.meletta@pharma.ethz.ch (R.M.); adrienne.herde@pharma.ethz.ch (A.M.H.); aristeidis.chiotellis@pharma.ethz.ch (A.C.); isam@student.ethz.ch (M.I.); simon.ametamey@pharma.ethz.ch (S.M.A.); roger.schibli@pharma.ethz.ch (R.S.); 2 Division of Cardiovascular Surgery, University Hospital Zurich, Rämistrasse 100, 8091 Zurich, Switzerland; E-Mail: zoran.rancic@usz.ch; 3 Institute for Veterinary Pathology, Vetsuisse Faculty, University of Zurich, Winterthurerstrasse 268, 8057 Zurich, Switzerland; E-Mail: n.borel@access.uzh.ch; 4 Center for Radiopharmaceutical Sciences ETH-PSI-USZ, Paul Scherrer Institute, 5232 Villigen PSI, Switzerland

**Keywords:** atherosclerosis, fibroblast activation protein, carotid artery plaque, boronic acid-based inhibitor

## Abstract

Research towards the non-invasive imaging of atherosclerotic plaques is of high clinical priority as early recognition of vulnerable plaques may reduce the incidence of cardiovascular events. The fibroblast activation protein alpha (FAP) was recently proposed as inflammation-induced protease involved in the process of plaque vulnerability. In this study, FAP mRNA and protein levels were investigated by quantitative polymerase chain reaction and immunohistochemistry, respectively, in human endarterectomized carotid plaques. A published boronic-acid based FAP inhibitor, MIP-1232, was synthetized and radiolabeled with iodine-125. The potential of this radiotracer to image plaques was evaluated by *in vitro* autoradiography with human carotid plaques. Specificity was assessed with a xenograft with high and one with low FAP level, grown in mice. Target expression analyses revealed a moderately higher protein level in atherosclerotic plaques than normal arteries correlating with plaque vulnerability. No difference in expression was determined on mRNA level. The radiotracer was successfully produced and accumulated strongly in the FAP-positive SK-Mel-187 melanoma xenograft *in vitro* while accumulation was negligible in an NCI-H69 xenograft with low FAP levels. Binding of the tracer to endarterectomized tissue was similar in plaques and normal arteries, hampering its use for atherosclerosis imaging.

## 1. Introduction

The concept of plaque vulnerability has changed the understanding of the pathogenesis of atherosclerosis and has led to novel perspectives for diagnostic and therapeutic interventions. The development of diagnostic methods to assess plaque vulnerability is considered an urgent priority in clinical and basic research [1]. The assessment of plaque vulnerability in patients at risk for cardiovascular disease would allow an adequate pharmacological and/or surgical treatment already in the asymptomatic stage and, therefore, reduce atherosclerosis-associated disability and mortality. Molecular imaging with suitable tracers has the potential to non-invasively identify molecular processes providing functional information about disease progression. In the asymptomatic stage, functional imaging may thus provide more specific information on plaque vulnerability than morphology-based imaging modalities [2]. Several imaging targets and the respective tracers are under investigation with the goal to image plaque vulnerability. The most prominent tracer is [^18^F]fluorodeoxyglucose, which accumulates in cells with high glucose consumption, including activated macrophages. However, the unspecific mechanisms of accumulation and the high uptake in myocardium limit its applicability [3].

Nowadays, plaque progression is regarded as a dynamic and complex process with stabilizing and destabilizing components involved. If destabilizing plaque components prevail over stabilizing factors an atherosclerotic plaque may eventually rupture leading to often severe or even fatal complications. Stabilizing components include an intact and thick fibrous cap that is formed by smooth muscle cells (SMCs) embedded in an extracellular matrix rich in collagen. On the contrary, plaque vulnerability is related to a thinning of the fibrous cap facilitated by the gradual loss of SMCs and the degradation of the collagen-rich fibrous cap [4]. The digestion of the extracellular matrix is caused by proteases in the atheromata which include matrix metalloproteinases (MMPs), cathepsins S/K and as recently proposed the fibroblast activation protein alpha (FAP, seprase) [5,6,7,8]. FAP is a type II membrane-bound serine protease belonging to the subfamily dipeptidyl peptidase IV N-terminal (DPP IV, S9B) within the prolyl oligopeptidase family (POP, S9) [9,10,11]. In contrast to other members of the DPP IV subfamily, FAP displays endo- besides exopeptidase activity [12]. FAP is capable of cleaving peptide bonds between proline and another amino acid [12]. FAP has gelatinase activity and is involved in the further digestion of degradation products of type I collagen [13,14,15,16]. The endo- and exopeptidase enzymatic activity requires homodimerization and glycosylation of the protease [10,14,17].

FAP was initially identified as a pivotal component of the tumor microenvironment expressed by reactive stromal fibroblasts in over 90% of common human epithelial carcinomas and may serve as a therapy target in oncology [18,19,20]. Furthermore, an association of FAP expression with inflammatory processes was described [18] and in line with this finding is emerging data by Brokopp *et al.* indicating an involvement of FAP in the pathogenesis of atherosclerosis [7]. In detail, Brokopp *et al.* showed that FAP is expressed by SMCs in human aortic plaques and confirmed its involvement in type I collagen degradation in aortic fibrous caps. Moreover, an association between tumor necrosis factor alpha (TNFα) secretion by macrophages with FAP expression in cultured human aortic SMCs and additionally a positive correlation of FAP-expressing SMCs with the macrophage burden in human aortic plaques was described [7]. The extent of FAP expression at different stages in atherosclerotic plaque progression was evaluated and revealed an increased FAP expression in advanced aortic plaques and in thin-cap *versus* thick-cap coronary atheromata by immunohistochemistry and immunofluorescence [7]. These findings indicate that FAP expression is related to plaque vulnerability with FAP representing an inflammation-induced protease in atherosclerosis. In this respect, FAP could serve as a promising target for non-invasive atherosclerotic plaque imaging. 

The goal of this study was to evaluate FAP as a target for atherosclerosis imaging with a small molecule. Imaging FAP density requires a FAP-selective ligand with high binding affinity. Several research groups have pursued to design small inhibitors with high specificity and selectivity towards individual serine proteases in the POP family. To selectively target FAP over other peptidases, its dual enzymatic activity as endo- and exopeptidase has to be considered. Identifying inhibitors with high selectivity for FAP over other DPPs and the most closely related prolyl endopeptidase PREP is challenging due to the 48% amino acid sequence identity of FAP and DPP-4, analogous substrate preferences and the ubiquitous expression of many proteases of the POP family [9,11]. Most FAP inhibitors share the pyrrolidine-2-boronic acid moiety as a common structural motif. The first boronic acid inhibitor reaching phase II clinical trials in the field of cancer treatment was ValboroPro (talabostat, PT-100), however due to missing selectivity clinical evaluation was terminated [21,22,23]. ValboroPro displayed IC_50_ values in the nanomolar range to several prolyl peptidases [24]. The introduction of a blocked N terminus in the dipeptidyl boronic acid structure led to novel inhibitors that were evaluated regarding binding affinity and selectivity [25,26,27,28,29] with the advantage of impeded intra-molecular cyclization reactions mediated by the electrophilic boron and an increased selectivity over DPPs that lack endopeptidase activity [30].

Marquis *et al.* presented a para-iodine substituted benzamido-glycine-boronoproline analog, MIP-1232, with an IC_50_ of 0.6 nM as determined in an enzyme inhibition assay with human recombinant FAP [29]. MIP-1232 was 32-fold more potent in inhibiting FAP than PREP. The corresponding K_d_ value of [^123^I]MIP-1232 in stably FAP-transfected human embryonic kidney cells (HEK-293) was 30 nM and different FAP-positive cell lines showed a markedly reduced enzymatic activity under MIP-1232 treatment compared to baseline conditions [29,31]. The high binding affinity to FAP and the selectivity profile in combination with the possibility to radioiodinate MIP-1232 without altering its structure make this compound a promising molecule to assess the potential of FAP as an imaging target for the staging of plaque vulnerability and to detect FAP-positive tumors that may respond to FAP-targeted therapy. In this study, we investigated FAP expression in human carotid specimens by quantitative polymerase chain reaction (qPCR) and immunohistochemistry (IHC). Furthermore, we synthesized MIP-1232 and subsequently radiolabeled this compound with iodine-125. Its accumulation in human atherosclerotic plaques was evaluated *in vitro* by autoradiography.

A FAP-positive SK-Mel-187 melanoma xenograft and an NCI-H69 xenograft with low FAP levels, both grown in mice, were used as controls.

## 2. Results and Discussion

### 2.1. Gene Expression Analysis of FAP and SMA in Human Carotid Plaques

**Figure 1:**
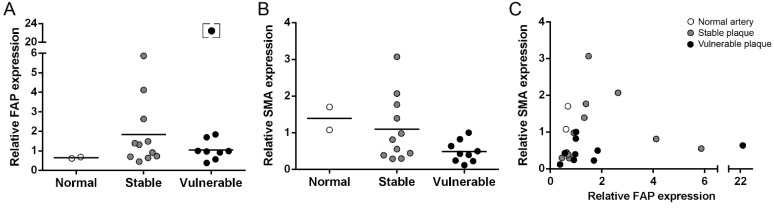
Relative mRNA expression levels of FAP (**A**) and SMA (**B**) in normal arteries (n = 2), stable plaques (n = 11) and vulnerable plaques (n = 9). For both proteins no significant difference was detected between stable and vulnerable plaques. (**C**) Comparison of the relative mRNA expression levels of FAP and SMA. mRNA expression was quantified by qPCR, shown are averages of three independent analyses. Lines indicate mean values. The square bracket indicates an outlier that was excluded from statistical analyses.

Quantitative expression analysis of FAP and alpha smooth muscle cell actin (SMA) by qPCR was performed with β-actin as reference gene (Figure 1). For FAP, a similar average gene expression was determined in normal arteries, stable plaques and vulnerable plaques (Figure 1A). The average SMA gene expression was not significantly different comparing vulnerable and stable plaques (Figure 1B). No significant correlation between the SMA and FAP gene expression in human endarterectomized plaques was observed (Figure 1C).

### 2.2. Immunohistochemical Staining of Human Carotid Plaques for FAP and SMA

**Figure 2:**
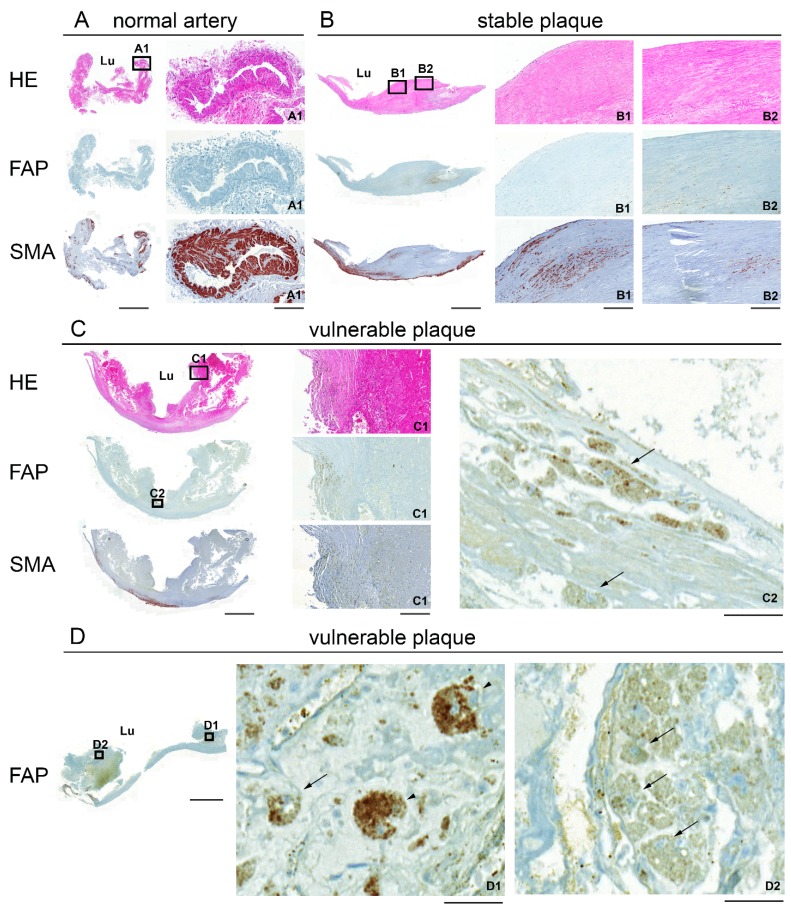
Hematoxylin/eosin (HE; **A**–**C**) and immunohistochemical (**A**–**D**) staining for FAP and SMA of representative 2 μm paraffin-embedded sections of a normal artery (A), a stable plaque (B) and vulnerable plaques (C,D). Boxed higher-magnification images show a small blood vessel (normal artery A1), regions in the fibrous cap (stable B1, B2 and vulnerable plaque C1) and FAP-positive macrophages (C2, arrows). (D) High magnification images show FAP-positive giant cells (D1, arrowheads) and macrophages (D1, D2, arrows) in a vulnerable plaque. The endarterectomized plaques are composed of tunica intima and part of the media. Lu: lumen. Scale bar, low magnification 2000 μm; A1, B1, B2, C1, 200 μm; C2, D1, D2, 50 μm.

The expression of FAP and SMA was further investigated by immunohistochemistry in consecutive sections of human atherosclerotic plaques (Figure 2). Normal arteries were FAP negative. In plaques, a focal FAP expression in macrophages and giant cells located in the superficial regions of the fibrous cap was observed with the most pronounced focal signals in vulnerable plaques (Figures 2C2,D1,D2). SMA was strongly expressed in the tunica media in all three classification categories with the highest expression in the vasa vasorum of normal arteries (Figure 2A1). The distribution pattern of SMA within atherosclerotic plaques was generally focal with major clusters in the cap or shoulder region. 

No distinct co-localization of the two expression markers was found in all examined carotid plaques (Figure 2B1,B2).

### 2.3. Chemistry and Radiochemistry

**Scheme 1:**
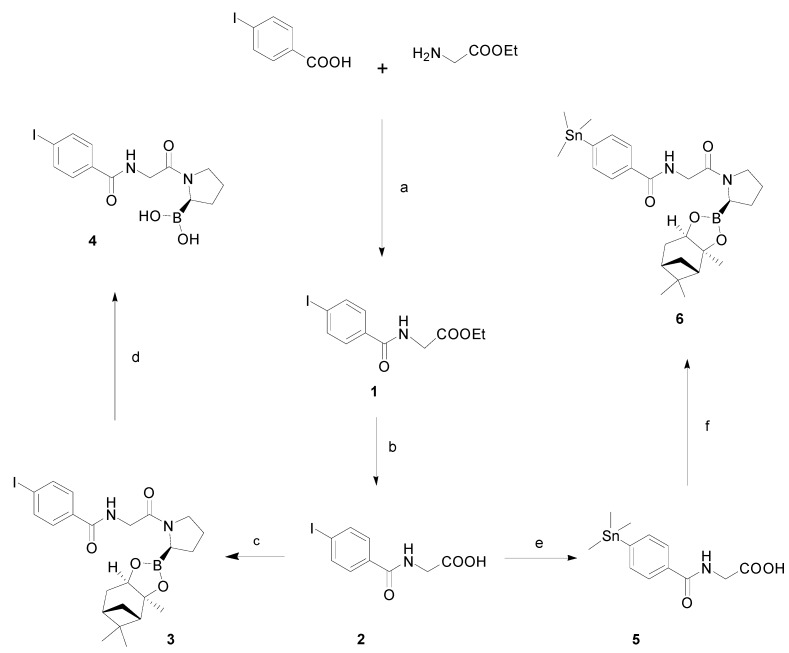
Synthesis of reference compound **4** and corresponding precursor **6**.

Notes: *Reagents and conditions:* (a) HBTU, DIPEA, DMF, rt, 3 h, 79%; (b) KOH, MeOH/H_2_O, rt, 1 h, 55%; (c) EDC, HOBt, (*R*)-BoroPro-(+)-pinanediol·HCl, DCM, 0 °C to rt, 16 h, 93%; (d) NH_4_OAc, NaIO_4_, acetone, rt, 17 h, 45%; (e) hexamethylditin, Pd(PPh_3_)_2_Cl_2_, dioxane, rt, 3 h, 81%; (f) EDC, HOBt, (*R*)-BoroPro-(+)-pinanediol·HCl, DCM, 0 °C to rt, 16 h, 59%.

Reference compound and precursor were synthesized from commercially available 4-iodobenzoic acid and glycine ethyl ester hydrochloride, as shown in Scheme 1. The synthetic scheme followed was the one reported by Zimmerman *et al.* [31] with some distinct modifications. For the reference compound, glycine ethyl ester was efficiently coupled to 4-iodobenzoic acid with HBTU as the coupling agent to afford compound **1** in 79% yield. The ethylester was then cleaved under basic conditions (aq. KOH/MeOH) to give the free acid **2** in moderate yield (55%) [32]. Reaction of compound **2** with (*R*)-boroPro-(+)-pinanediol·HCl using the EDC/HOBt coupling system afforded dipeptide **3** in excellent yield (93%). 

Deprotection of the boronic ester to the free boronic acid, proved challenging. The proposed transesterfication method [31] with phenylboronic acid was not efficient in our hands. Apart from solubility problems the reaction was sluggish producing many byproducts. Thus, an alternative method was applied, which involved oxidative cleavage of the pinanediol protecting group using sodium metaperiodate [33]. This procedure was more compatible with our substrate, yielding cleanly and relatively fast a new more polar product as revealed by HPLC monitoring. After workup, the crude was purified with preparative RP-HPLC to provide **4** in moderate yield (45%).

For the synthesis of the precursor, a similar procedure was followed. Stannylation of the iodinated compound **2** was achieved by reacting it with hexamethylditin and Pd(PPh_3_)_2_Cl_2_ in refluxing dioxane, which yielded compound **5** in a good yield (81%). **5** was then coupled to the boronic ester (*R*)-boroPro-(+)-pinanediol with EDC/HOBt in DCM and the crude was purified with RP-HPLC to provide the precursor **6** in satisfactory yield (59%).

**Scheme 2:**
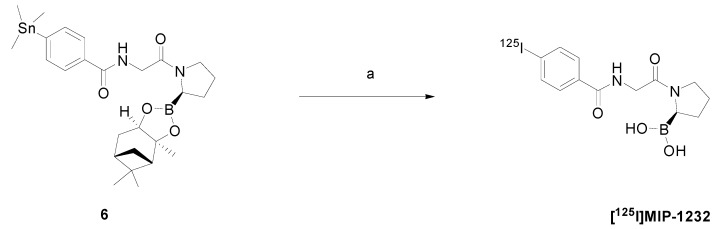
Radioiodination scheme of precursor **6** to [^125^I]MIP-1232.

Note: *Reagents and conditions:* Na[^125^I], H_2_O_2_, H_2_SO_4_, CH_3_COOH, MeCN, rt, 10 min then Na_2_S_2_O_3_.

[^125^I]MIP-1232 was produced in a one-step reaction by electrophilic radioiodination of the corresponding trimethylstannyl precursor (Scheme 2). The procedure was performed according to Zimmerman *et al.* [31] with some modifications since the order of addition of the reagents critically affected the outcome of the reaction. The experimental protocol was optimized so as to yield a reliable and robust radiolabeling procedure. Briefly, precursor **6** was incubated with Na[^125^I] under oxidative conditions to achieve electrophilic radioiodination and simultaneously cleaving the boronic acid protecting group. After quenching with Na_2_S_2_O_3_, the reaction mixture was purified by analytical HPLC to yield [^125^I]MIP-1232 in 10%–12% radiochemical yield (decay-corrected; n = 3) and radiochemical purity ≥ 90%. 

The stability in acetonitrile/water/TFA (as eluted from the HPLC column) was investigated by HPLC with reference compound **4**. The compound was stable with >96% intact compound present after 110 h storage. [^125^I]MIP-1232 was stored under identical conditions and all experiments were performed within this time period after purification. 

### 2.4. In Vitro Autoradiography

**Figure 3:**
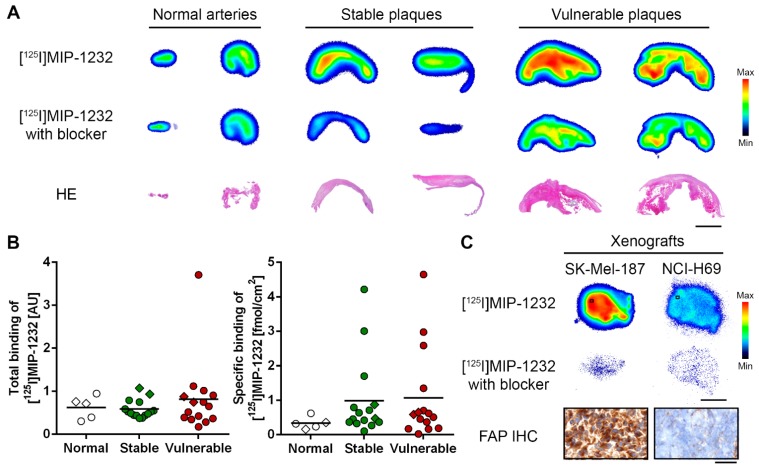
(**A**) *In vitro* autoradiogram of representative sections of human carotid plaques under baseline ([^125^I]MIP-1232) and blockade condition ([^125^I]MIP-1232 with excess unlabeled MIP-1232). Hematoxylin/eosin (HE) staining below represents plaque morphology. Scale bar 3 mm. (**B**) Quantified total and specific binding of [^125^I]MIP-1232 to normal arteries (n = 5), stable plaques (n = 16) and vulnerable plaques (n = 15) determined by autoradiography and corrected for tissue size. No significant intergroup differences were determined. Lines indicate mean values, diamonds indicate the specimens shown in A. (**C**) *In vitro* autoradiography with xenografts under baseline and blockade conditions. IHC staining for FAP of the SK-Mel-187 and the NCI-H69 xenograft (20 µm cryosections). Scale bar 3 mm for autoradiography; 50 µm for IHC images. Color scales for minimal to maximal binding.

Radiotracer binding was evaluated by *in vitro* autoradiography with human carotid plaques and xenograft tissue, as shown in Figure 3. [^125^I]MIP-1232 binding was higher in atherosclerotic plaques than normal arteries. Vulnerable plaques showed a slightly higher radioactivity signal integrated over the tissue slice than stable plaques. However, after correction for the size of the tissue samples, average total binding was similar for the three categories (Figure 3A,B). Radiotracer binding was reduced under blockade conditions with an excess of unlabeled MIP-1232 indicating displaceable (specific) binding of [^125^I]MIP-1232 (Figure 3A). No significant difference was detected comparing the specific binding of the three groups (Figure 3B). In a proof-of-principle study, target specificity of [^125^I]MIP-1232 was evaluated in an autoradiography assay with xenograft tissue (Figure 3C). FAP-positive SK-Mel-187 melanoma xenografts [34] displayed a markedly higher radioactivity signal than NCI-H69 lung small cell carcinoma xenografts and radiotracer binding was blocked completely by excess of MIP-1232 in both xenografts. IHC experiments confirmed high FAP levels in the SK-Mel-187 xenograft and low levels in the NCI-H69 xenograft (Figure 3C).

### 2.5. Discussion

Recent studies suggest that inflammation-related processes provide promising targets for the non-invasive imaging of plaque vulnerability [35,36,37]. We lately identified the co-stimulatory molecule CD80 involved in T cell activation as a promising imaging target since its expression is increased in vulnerable plaques. A radiolabeled specific inhibitor accumulated in human vulnerable plaques *in vitro* [38]. Moreover, we evaluated an F-18 labeled folate derivative targeting activated macrophages that accumulated stronger in atherosclerotic plaques than normal arteries [39]. Brokopp *et al.* presented another inflammation-related target, FAP, that displayed increased levels in advanced plaques indicating an association with the process of plaque destabilization [7]. The mechanistic role of FAP in atherosclerosis remains vague. Collagen, thereof 70% collagen type I, is a primary component of the extracellular matrix in atherosclerotic plaques [40]. Synergistically with matrix metalloproteinases, FAP is capable of degrading type I collagen and these proteases therefore have a destabilizing effect on atherosclerotic plaques [15]. The involvement of serine proteases, in particular of the DPP IV subfamily, in atherosclerosis and its clinical adverse events certainly warrants further investigations.

In this study, we analyzed plaque specimens obtained from the carotid artery that showed similar FAP mRNA levels as normal artery segments, independent of plaque vulnerability. In agreement with Brokopp *et al.*, FAP protein levels as determined by IHC correlated with plaque progression, with the highest focal staining in vulnerable plaques. The discrepancy between mRNA and protein levels could indicate lower degradation of FAP in vulnerable than stable plaques, in line with differences in protease and protease inhibitor levels in the two lesion types [8]. Overall, the difference in FAP expression between normal arteries and plaques was modest in our study. In contrast to the publication of Brokopp *et al.*, we found FAP protein in macrophages and giant cells within the plaque and found no co-localization with SMCs. However, we want to point out that we evaluated artery segments of a different location and used different tissue preparations than Brokopp and colleagues. The localization of FAP in macrophages in our study is in agreement with recent reports showing FAP expression in M2 macrophages [41,42]. 

The FAP inhibitor MIP-1232 was successfully synthesized and radiolabeled with iodine-125, a long-lived gamma ray-emitting nuclide. The synthesis and radiolabeling were accomplished in reasonable yields and purity. In a proof-of-principle study with a FAP-positive SK-Mel-187 xenograft [34], a high and displaceable binding of the radiotracer was observed, whereas binding to the NCI-H69 xenograft with low FAP levels was negligible. This indicates binding of [^125^I]MIP-1232 to FAP-positive tissue *in vitro*.

The potential of this radiotracer for atherosclerotic plaque imaging was investigated by *in vitro* autoradiography with human carotid plaques. Here, we found a pronounced binding to carotid plaques, however with no difference in average specific binding between stable and vulnerable plaques and between plaques and normal arteries, after correction for the size of the tissue samples. However, 3 of the 16 stable and 4 of the 15 vulnerable plaques showed several-fold higher specific accumulation than normal arteries. Only a prospective study would show whether this is of clinical relevance.

Based on our data we cannot conclude on the selectivity of [^125^I]MIP-1232 for FAP. In the absence of a known selective inhibitor, we investigated specificity by blocking with the unlabeled compound itself. The relatively high amount of remaining radiotracer after blocking must, therefore, accumulate with low affinity. Lipophilicity is most probably not involved as clogP of MIP-1232 is about 0.5. The non-specific accumulation may result from interactions with highly abundant hydrolases or other proteins with affinities in the high micromolar range, considering that our blocker concentration was 100 µM. Specificity analysis of MIP-1232 was performed exclusively with FAP and PREP [29]. A conclusive evaluation of the binding affinity to dipeptidyl peptidases such as DPP-2, DPP-4, DPP-8 and DPP-9 would be required, irrespective of the fact that DPPs display in general low affinities for N blocked peptides [43,44]. As [^125^I]MIP-1232 did not selectively accumulate in the atherosclerotic tissue and as its low FAP/PREP affinity ratio is already known we did not further investigate its selectivity profile. For future studies more selective inhibitors are needed to reduce non-specific tissue accumulation. To overcome limitations in specificity, novel lead structures and the use of antibodies and fusion proteins was proposed to minimize off-target effects [24,34,44]. 

Our findings with the tumor xenografts are of interest in oncology [34,45,46]. Although FAP as a target may be of little relevance for tumor imaging in general considering the high diagnostic value of [^18^F]fluorodeoxyglucose; FAP imaging with a selective ligand would enable the identification of FAP-positive tumors sensitive to a FAP-targeted radiotherapy with existing antibodies [34].

## 3. Experimental Section 

### 3.1. Patient Characteristics and Human Carotid Tissue Banking

Human atherosclerotic plaque tissues were excised during carotid endarterectomy (CEA) surgery at the University Hospital Zurich using the bifurcation advancement technique [47]. The atherosclerotic material was removed from the common, external and internal carotid artery. Before surgery, written informed consent was obtained from all patients. A total of 25 patients were included in this study with an average age of 73.1 years (73.1 ± 6.6 y) at surgery and 84% of them male. After CEA, the tissue was transferred to RNAlater^®^ solution (Sigma, St. Louis, MO, USA) and stored at −80 °C until further use. Excised material was classified into the categories “normal artery”, “stable plaque” and “vulnerable plaque” based on a macroscopic visual inspection and a histological examination. The histological analysis was performed with standard staining methods (e.g., hematoxylin and eosin) and according to the classification system of the American Heart Association as previously described [38,48]. Plaques were classified as stable if there was a lipid core separated to the blood stream by an intact fibrous cap with a representative cap thickness >500 μm and a minimum cap thickness >200 μm [49]. Vulnerable plaques were lesions with a large necrotic core, a thin or ruptured fibrous cap, high infiltration of inflammatory cells and neovascularization. The microscopic characterization of all plaques used in this study was in agreement with the macroscopic evaluation. In total 7 normal arteries, 25 stable plaques and 23 vulnerable plaques were used for gene expression analysis (normal n = 2, stable n = 11, vulnerable n = 9), immunohistochemistry (normal n = 1, stable n = 4, vulnerable n = 4) and autoradiography (normal n = 5, stable n = 16, vulnerable n = 15), respectively. Classified normal arteries were redundant segments from the *A. iliaca* or *A. thyroidea*. Note the limited availability of normal arteries.

### 3.2. RNA Isolation, Reverse-Transcription and Real-Time Polymerase Chain Reaction

Total RNA was isolated from human atherosclerotic plaque segments according to the protocol of the Isol-RNA Lysis reagent (5 PRIME, Gaithersburg, MD, USA) using a TissueLyser bead-mill system (Qiagen, Hilden, Germany). cDNA was generated by the QuantiTect Reverse Transcription Kit (Qiagen, Hilden, Germany). Used primers were custom-made oligonucleotides from Microsynth (Balgach, Switzerland): human actin β (ACTB) (forward 5'-CATGTACGTTGCTATCCAGGC-3', reverse 5'-CTCCTTAATGTCACGCACGAT-3', NM_001101), human fibroblast activation protein alpha (FAP) (forward 5'-TGAA CGAGTATGTTTGCAGTGG-3', reverse 5'-GGTCTTTGGACAATCCCATGT-3', NM_004460) and human alpha smooth muscle cell actin (SMA) (forward 5'-GCTGGCATCCATGAAACCAC-3', reverse 5'-TGCCCCCTGATAGGACATTG-3', NM_001613). Quantitative polymerase chain reaction (qPCR) was performed with the DyNAmo^TM^ Flash SYBR^®^ Green PCR System (Applied Biosystems, Foster City, CA, USA) using a AB7900 HT Fast Real-Time PCR System (Applied Biosystems). Quantification was performed by the 2^−∆∆Ct^ quantification method with β-actin as a reference gene [50]. All reactions were conducted in duplicates in three independent experiments. Specificity of the amplification products was assured by dissociation analysis. SMA is specifically expressed in SMCs of different origin.

### 3.3. Histology and Immunohistochemistry

Plaques were paraffin-embedded and serial sections of 2 μm were prepared for further histological and immunohistochemical investigations. Hematoxylin and eosin (HE) staining was performed according to routine procedure to classify plaques into the categories “stable” and “vulnerable”. For immunohistochemistry, primary antibodies for FAP (anti-FAP, 1:50, rabbit, polyclonal antibody directed against the Fibroblast activation protein, NB100-91763, Novus Biologicals (Littleton, CO, USA) and SMA (anti-SMA, 1:400, mouse, monoclonal antibody directed against anti-human alpha smooth muscle actin, M0851, Dako, Baar, ZG, Switzerland) were used. Antigen retrieval for the anti-FAP antibody was performed using acid buffer (pH 6.0), whereas no antigen retrieval was performed for the anti-SMA antibody. The detection system included the OmniUltraMab Kit (Roche, Rotkreuz, ZG, Switzerland) for the anti-FAP antibody on the Discovery XT instrument (Roche) and the Dako RealKit (Dako) for the anti-SMA antibody on the immunostainer (Dako). FAP IHC staining of a SK-Mel-187 and a NCI-H69 xenograft was performed with 20 µm frozen sections according to the above specified procedure without antigen retrieval. Sections were scanned by a slide scanner (Pannoramic 250, 3D Histech, Sysmex, Horgen, Switzerland). HE and IHC staining were analyzed by a pathologist (N.B.).

### 3.4. Chemicals and Reagents

All reagents and starting materials were purchased from commercial suppliers and used without further purification. All solvents used for reactions were obtained as anhydrous grade (puriss., dried over molecular sieves, H_2_O <0.005%) from Acros Organics (Geel, Belgium) and were used without further purification unless otherwise stated. Solvents for extractions, column chromatography and thin layer chromatography (TLC) were purchased as commercial grade. All non-aqueous reactions were performed under an argon atmosphere using flame-dried glassware and standard syringe/septa techniques. In general, reactions were magnetically stirred and monitored by TLC performed on Merck (Merck Millipore, Schaffhausen, Switzerland) TLC glass sheets (silica gel 60 F_254_). Spots were visualized with UV light (λ = 254 nm) or through staining with anisaldehyde solution or basic aq. KMnO_4_ solution and subsequent heating. Chromatographic purification of products was performed using silica gel 60 for preparative column chromatography (particle size 40–63 µm, Fluka, Buchs, Switzerland). Reactions at 0 °C were carried out in an ice/water bath. Nuclear magnetic resonance (NMR) spectra were recorded in CDCl_3_, CD_3_OD or DMSO-*d*_6_ on an AV-400 spectrometer (Bruker, Billerica, MA, USA) at room temperature. The measured chemical shifts are reported in δ (ppm) and the residual signal of the solvent was used as the internal standard (CDCl_3_^1^H: δ = 7.26 ppm, ^13^C: δ = 77.0 ppm; CD_3_OD ^1^H: δ = 3.31 ppm, ^13^C: δ = 49.15 ppm; DMSO-*d*_6_^1^H: δ = 2.50 ppm, ^13^C: δ = 39.51 ppm). All ^13^C-NMR spectra were measured with complete proton decoupling. Data of NMR spectra are reported as follows: s = singlet, d = doublet, t = triplet, q = quartet, m = multiplet, dd = doublet of doublets, dt = doublet of triplets, br = broad signal. The coupling constant *J* is reported in Hertz (Hz). Electrospray (ES) mass spectra (HRMS) were obtained with a Bruker’s maXis (ESI-Qq-TOF-MS) spectrometer. Analytical HPLC was performed with a reverse phase column (Ultimate^®^ XB-C18 column 4.5 × 250 mm, 5 µm) with the following solvent system: water/0.1% TFA (solvent A), acetonitrile (solvent B); 0–30 min: 25% B (system 1) or 0–30 min: 75% B (system 2). The flow rate was 1 mL/min and UV detection at 254 nm. Preparative HPLC was performed with a reverse phase preparative column (Ultimate^®^ XB-C18 column 21.2 × 150 mm, 5 µm) using the above mentioned isocratic conditions for analytical HPLC at a flow of 20 mL/min and UV detection at 254 nm.

*In vitro* stability evaluation of the reference compound (**4**) in formulation (30% acetonitrile, 70% water/0.1% TFA) was performed with a reverse phase column (LUNA^®^ Phenomenex C18 4.5 × 250 mm, 5 µm). The following solvent system was applied: water/0.1% TFA (solvent A), acetonitrile (solvent B); flow 1 mL/min; 0–13 min: 30% B, 13–18 min: 30%–80% B, 18–36 min: 80% B, 36–38 min: 80%–30% B, 38–40 min: 30% B; UV = 254 nm. Stability was assessed up to 110 h. 

Purification and analytics of the radiolabeled material was performed on a Merck Hitachi D-6000 system (Merck Hitachi, San Jose, CA, USA) equipped with multi-UV-wavelength and Raytest Gabi Star detectors and HSM software. A reverse phase column was used (LUNA^®^ Phenomenex C18 4.5 × 250 mm, 5 µm) according to the above specified solvent system and conditions for the stability evaluation. 

### 3.5. Chemistry

*Ethyl 2-(4-iodobenzamido)acetate* (**1**). To a solution of 4-iodobenzoic acid (1.5 g, 6.05 mmol) and *N*,*N*-diisopropylethylamine (2.1 mL, 12.1 mmol) in DMF (23.6 mL), *O*-(Benzotriazol-1-y)-*N*,*N*,*N'*,*N'*-tetramethyluronium hexafluorophosphate (2.75 g, 7.26 mmol) was added portionwise at room temperature. After stirring for 15 min, a solution of glycine ethyl ester hydrochloride (0.748 g, 7.26 mmol) and *N*,*N*-diisopropylethylamine (2.1 mL, 12.1 mmol) in DMF (10 mL) was added dropwise and the reaction mixture was stirred for 3 h. The mixture was then diluted with ethyl acetate and washed successively with 0.5 M HCl, 5% NaHCO_3_, H_2_O and brine. The organic layer was dried over MgSO_4_ and concentrated *in vacuo*. After evaporating, the residue was purified by flash column chromatography on silica gel (hexane/AcOEt 7:3) to afford compound (**1**) (1.59 g, 79%) as light yellow solid. NMR data were in accordance with previously published data [32]. R*f*: 0.24 (hexane/EtOAc 7:3). ^1^H-NMR (400 Hz, CDCl_3_): δ = 7.82–7.77 (m, 2H), 7.56–7.50 (m, 2H), 6.68 (br, 1H), 4.26 (q, *J* = 7.2 Hz, 2H), 4.21 (d, *J* = 4.9 Hz, 2H), 1.31 (t, *J* = 7.2 Hz, 3H).

*2-(4-Iodobenzamido)acetic acid* (**2**). To a solution of ethyl 2-(4-iodobenzamido)acetate (1.4 g, 4.2 mmol) in MeOH (35 mL) and H_2_O (35 mL), KOH (707 mg, 12.61 mmol) was added with continuous stirring and was kept for 1 h at room temperature, at which point TLC confirmed the complete consumption of the starting material (**1**). The reaction was then diluted with H_2_O (42 mL) and the pH of the solution was adjusted to 2 with HCl 1 M. The precipitate was filtered off and washed with cold H_2_O. The compound was dried under high vacuum over P_2_O_5_ for 3 h to afford (**2**) as a white solid (707 mg, 55%). R*f*: 0.48 (DCM/MeOH/AcOH 9:1:0.02). ^1^H-NMR (400 Hz, DMSO): δ = 8.88 (t, *J* = 5.6 Hz, 1H), 7.89–7.85 (m, 2H), 7.67–7.62 (m, 2H), 3.89 (d, *J* = 5.6 Hz, 2H). ^13^C-NMR (100 Hz, DMSO): δ = 171.2, 165.7, 137.2, 133.3, 129.2, 99.1, 41.3.

*4-Iodo-N-(2-oxo-2-((R)-2-((3aS,4S,6S,7aR)-3a,5,5-trimethylhexahydro-4,6-methanobenzo[d][1,3,2]-dioxaborol-2-yl)pyrrolidin-1-yl)ethyl)benzamide* (**3**). To an ice-cooled solution of 2-(4-iodo-benzamido)acetic acid (300 mg, 0.983 mmol) in DCM (5.6 mL), hydroxybenzotriazole (151 mg, 0.98 mmol) was added, followed by 1-(3-dimethylaminopropyl)-3-ethylcarbodiimide hydrochloride (EDCI) (245 mg, 1.28 mmol) and the resulting mixture was stirred at room temperature for 30 min. *N*-Methylmorpholine (0.2 mL, 1.967 mmol) and (*R*)-boroPro-(+)-pinanediol·HCl (245 mg, 0.98 mmol) were then added and stirring was continued for 16 h. The reaction mixture was diluted with DCM, washed successively with 1 M KHSO_4_, 10% Na_2_CO_3_, H_2_O and brine. The organic layer was dried over MgSO_4_, filtered and concentrated *in vacuo*. After evaporating, the residue was purified by passing quickly through a short plug of silica gel, eluting with EtOAc, to afford compound **3** (489 mg, 93%). R*f*: 0.52 (hexane/EtOAc 3:7). ^1^H-NMR (400 Hz, CDCl_3_): δ = 7.82–7.77 (m, 2H), 7.59–7.53 (m, 2H), 6.65 (br, 1H), 4.33 (br, 1H), 4.15 (d, *J* = 3.8 Hz, 2H), 3.51–3.41 (m, 2H), 3.24–3.17 (m, 1H), 2.39–2.30 (m, 1H), 2.22–1.96 (m, 5H), 1.94–1.78 (m, 4H), 1,46 (s, 3H), 1.29 (br, 3H), 0.84 (s, 3H). ^13^C-NMR (100 Hz, CDCl_3_): δ = 166.5, 166.1, 137.9, 133.7, 128.9, 128.5, 98.8, 86.4, 78.1, 51.5, 45.9, 42.5, 39.7, 38.5, 35.7, 28.7, 27.6, 27.3, 27.2, 26.5, 24.3. ESI-QTOF MS (DCM/MeOH) *m/z* calculated for C_23_H_31_BIN_2_O_4_ [M+H]^+^ 537.1420, measured 537.1414.

*(R)-(1-((4-Iodobenzoyl)glycyl)pyrrolidin-2-yl)boronic acid* (**4**). To a stirred solution of **3** (150 mg, 0.28 mmol) in acetone (7.4 mL) was added 0.1 M NH_4_OAc (6 mL, 0.60 mmol) and NaIO_4_ (189 mg, 0.88 mmol). The mixture was stirred at room temperature for 17 h, the acetone was removed *in vacuo* and the aqueous phase was turned basic with 2 M NaOH (9 mL), washed with DCM and acidified cautiously to pH 2 with 2M HCl. The acidic solution was extracted with DCM (4x), dried over MgSO_4_, filtered and evaporated to dryness. The crude was purified with preparative HPLC using system 1 to afford compound **4** (45 mg, 45%). R*f*: 0.19 (EtOAc). ^1^H-NMR (400 Hz, MeOD): δ = 7.88–7.83 (m, 2H), 7.65–7.59 (m, 2H), 4.42–4.08 (m, 2H), 3.69–3.48 (m, 2H), 3.15–3.07 (m, 1H), 2.22–1.86 (m, 4H), 1.76–1.63 (m, 2H). ^13^C-NMR (100 Hz, MeOD): δ = 139.1, 130.3, 129.5, 47.0, 46.8, 43.1, 41.9, 36.5, 28.8, 27.6, 21.9. HRMS *m/z* calculated for C_14_H_17_BIN_2_O_3_ [M+H^+^−H_2_O+CH_2_] 399.0377, measured 399.0365.

*2-(4-(Trimethylstannyl)benzamido)acetic acid* (**5**). To a solution of 2-(4-iodobenzamido)acetic acid (331 mg, 1.085 mmol) in dry dioxane (6.7 mL), hexamethylditin (0.4 mL, 1.93 mmol) was added, followed by Pd(PPh_3_)_2_Cl_2_ (43.5 mg, 0.062 mmol) and the reaction mixture was heated under reflux for 3 h. After this time, the mixture was filtered through a pad Celite and the solvent was removed under reduced pressure. The crude was purified by flash column chromatography on silica gel (hexane/EtOAc/AcOH 60:40:0.4) to afford **5** (301 mg, 81%) as a clear colorless oil [18]. R*f*: 0.21 (hexane/EtOAc/AcOH 60:40:0.4). ^1^H-NMR (400 Hz, MeOD): δ = 7.81–7.77 (m, 2H), 7.61–7.57 (m, 2H), 4.01 (s, 2H), 0.37–0.21 (br, 9H). ^13^C-NMR (100 Hz, MeOD): δ = 172.4, 170.1, 148.5, 136.5, 134.6, 127.2, 43.0, −2.2. MALDI MS (3-HPA) *m/z* calculated for C_12_H_18_NO_3_Sn [M+H]^+^ 344.0305, measured 344.0304.

*N-(2-Oxo-2-((R)-2-((3aS,4S,6S,7aR)-3a,5,5-trimethylhexahydro-4,6-methanobenzo[d][1,3,2]dioxabo-rol-2-yl)pyrrolidin-1-yl)ethyl)-4-(trimethylstannyl)benzamide* (**6**). To an ice-cooled solution of 2-(4-(trimethylstannyl)benzamido)acetic acid (220 mg, 0.643 mmol) in DCM (3.7 mL), hydroxybenzotriazole (99 mg, 0.64 mmol) was added followed by EDCI (160 mg, 0.84 mmol) with continous stirring and was kept for 30 min at room temperature. N-Methylmorpholine (0.15 mL, 1.287 mmol) and (*R*)-boroPro-(+)-pinanediol·HCl (160 mg, 0.64 mmol) were then added and the reaction mixture was stirred for 16 h. The reaction mixture was diluted with DCM and washed with 1 M KHSO_4_, 10% Na_2_CO_3_, H_2_O and brine. The organic layer was dried over MgSO_4_, filtered and concentrated *in vacuo*. The crude was purified with preparative HPLC using system 2 to afford compound (**6**) (216 mg, 59%) as a white solid. R*f*: 0.56 (AcOEt). ^1^H-NMR (400 Hz, CDCl_3_): δ = 7.80–7.76 (m, 2H), 7.58–7.54 (m, 2H), 6.64 (br, 1H), 4.33 (br, 1H), 4.18 (d, *J* = 3.5 Hz, 2H), 3.54–3.41 (m, 2H), 3.24–3.18 (m, 1H), 2.37–2.29 (m, 1H), 2.22–2.08 (m, 5H), 1.94–1.77 (m, 4H), 1,46 (s, 3H), 1.29 (s, 3H), 0.85 (s, 3H), 0.39–0.23 (s, 9H). ^13^C-NMR (100 Hz, CDCl_3_): δ =167.5, 166.4, 136.2, 132.3, 128.6, 126.5, 98.7, 86.4, 78.1, 51.5, 45.9, 42.5, 39.8, 38.5, 35.7, 28.6, 27.6, 27.4, 27.2, 26.5, 24.3, −9.3. ESI-QTOF MS *m/z* calculated for C_26_H_40_BN_2_O_4_Sn [M+H]^+^ 575.2106, measured 575.2102.

### 3.6. Radiochemistry

MeCN (500 µL), 50% H_2_SO_4_ (50 µL) and freshly prepared oxidant (100 µL, 4% CH_3_COOH and 6.7% H_2_O_2_) were added to a sealed reaction vial. Na[^125^I] (1.3–34.6 MBq, Perkin Elmer, Waltham, MA, USA) was diluted with water (*ad* 50 µL) and added simultaneously with the precursor (100 μL, 1 mg/mL in MeCN) to the reaction mixture. The mixture was incubated for 10 min at room temperature with intermittent shaking. After this time, the reaction was quenched by the addition of 0.1 M Na_2_S_2_O_3_ (200 µL). The product was purified by analytical RP-HPLC. The purified product was obtained in 30% acetonitrile and 70% water/0.1% TFA and was stored at 4 °C until *in vitro* experimentation. Product identification was confirmed by co-injection of the reference **4**. Radiochemical purity was determined by analytical HPLC.

### 3.7. In Vitro Autoradiography

For *in vitro* autoradiography, cryosections and paraffin-embedded sections of human atherosclerotic plaques (20 µm and 5 μm, respectively) and paraffin-embedded sections (5 μm) of a FAP-positive melanoma xenograft (human skin melanoma cell line SK-Mel-187) and a human lung small cell carcinoma cell line NCI-H69 xenograft were used. A SK-Mel-187 xenograft was kindly provided by Dr. E. Fischer (Paul Scherrer Institut, Villigen, Switzerland). Cryosections were thawed and dried at room temperature for 30 min and all sections were subsequently incubated in HEPES buffer (50 mM HEPES, 5 mM MgCl_2_, 125 mM CaCl_2_, 0.1% BSA, pH 7.4) for 15 min on ice. The slices were incubated with [^125^I]MIP-1232 solution (2.95 nM in HEPES buffer, 0.1% BSA) or for blockade conditions with [^125^I]MIP-1232 solution containing additionally 100 µM unlabeled MIP-1232 for 60 min at room temperature in a humidified chamber. After incubation, the slices were washed in HEPES buffer supplemented with 0.1% BSA (5 min), three times in HEPES buffer (3 min each) and distilled water (1 min) on ice. For quantification of the radiotracer signal a calibration curve of a serial dilution of the tracer solution on Whatman filter paper (Whatman, Bottmingen, Switzerland) was used. Dried slides and the filter papers were exposed to a BAS-MS 2025 phosphor imaging plate (Fuji Film, Dielsdorf, Switzerland) for 19 h. The plate was scanned in a BAS-5000 bio-imaging analyzer (Fuji Film). Data analysis and quantification was performed with the AIDA 4.5 software (Raytest, Sprockhövel, Germany). Background values were subtracted from sample values and it was assured that all samples were within the linear range of the calibration curve. The spatially integrated signal intensities were divided by the plaque size to correct for heterogeneity in tissue size. Displaceable binding was calculated by subtraction of the radioactivity signal under blockade conditions from the baseline signal. 

### 3.8. Statistical Analysis

Differences in mean values were evaluated by an unpaired two-tailed student’s *t*-test (GraphPad Prism 6.0 software, GraphPad, La Jolla, CA, USA). A *p*-value < 0.05 was considered significant. 

## 4. Conclusions 

Target expression analysis by IHC revealed moderately higher levels of FAP in plaques than normal arteries. The radiolabeled boronic acid-based inhibitor, [^125^I]MIP-1232, was successfully produced. The radiotracer displayed displaceable binding to FAP-positive xenografts *in vitro* and accumulation in human carotid plaques *in vitro*. However, binding was similar in plaques and normal arteries and was independent of plaque vulnerability. Targeting FAP by [^125^I]MIP-1232 may, therefore, be of low relevance for atherosclerosis imaging. The high binding of [^125^I]MIP-1232 to a FAP-positive SK-Mel-187 xenograft but low binding to a xenograft with low FAP levels is promising towards the imaging of FAP to support FAP-targeted therapy in oncology.
